# Is migraine a spectrum disorder? Questioning the concept of “episodic” and “chronic” migraine using population-based data from 10,226 adults with migraine from 14 countries

**DOI:** 10.1186/s10194-026-02440-w

**Published:** 2026-06-26

**Authors:** Andreas Kattem Husøy, Timothy J. Steiner

**Affiliations:** 1 NorHead, Department of Neuromedicine and Movement Science, NTNU, Norwegian University of Science and Technology, Edvard Griegs gate, Trondheim, Norway; 2 Department of Neurology and Clinical Neurophysiology, St Olavs University Hospital, Trondheim, Norway; 3 Department of Neurology, University of Copenhagen, Copenhagen, Denmark; 4 Division of Brain Sciences, Imperial College London, London, UK

**Keywords:** Migraine, Chronic migraine, Episodic migraine, High-frequency episodic migraine, Medication-overuse headache, Meta-analysis, Individual participant data, Population-based study, HARDSHIP questionnaire, International Classification of Headache Disorders, Global Campaign against Headache

## Abstract

**Background:**

The first edition of the International Classification of Headache Disorders (ICHD-I) did not recognize chronic migraine (CM). ICHD-II introduced CM as a complication of migraine, with criteria that were widely criticized and later revised to require ≥ 15 monthly headache days (MHDs) with ≥ 8 monthly migraine days (MMDs). ICHD-3 reclassified CM as a type distinct from episodic migraine, retaining these frequency thresholds but without empirical justification. We investigated whether population-based evidence on headache characteristics and burden supported distinction between episodic and chronic migraine types, or indicated that migraine was better conceptualized as a single disorder expressed along a frequency spectrum.

**Methods:**

We performed a meta-analysis of individual participant data from 15 cross-sectional population-based surveys from the Global Campaign against Headache, all randomly selecting adults (18–65 years) and using the HARDSHIP questionnaire for data collection. Diagnoses made algorithmically observed the hierarchical order of ICHD. We identified four migraine groups: (1) migraine (definite+probable) including, and (2) migraine (definite+probable) excluding those with concomitant probable medication-overuse headache (pMOH); (3) migraine (definite only) including, and (4) migraine (definite only) excluding those with concomitant pMOH. MMDs and MHDs were regressed against headache characteristics, quality of life (QoL) and impaired participation.

**Results:**

Among 36,407 participants (mean age 37.5 years; 53.4% females), 10,266 (28.2%) met criteria for migraine (14.6% definite; 13.6% probable), including 896 (2.5%) with concomitant pMOH. Distributions of MMDs and MHDs were heavily right-skewed, peaking at 3 days/month, but showed no inflections at the thresholds for CM. Age was positively associated with headache frequency, while most headache characteristics varied minimally across frequencies. QoL declined slightly but linearly with increasing frequency, whereas lost workdays, household days and social days increased more-or-less linearly (albeit that household days plateaued after 15 days/3 months). Regressions were strikingly similar across genders and the four migraine groups.

**Conclusion:**

Population-based evidence indicates that migraine, considered in relation to headache frequency, characteristics and attributed burden, is better understood as a spectrum disorder rather than as two (or more) entities distinguished categorically by frequency. The current thresholds for CM do not align with detectable step-increments in headache characteristics or burden. These findings fill a major knowledge gap, usefully informing the development of ICHD-4.

**Supplementary Information:**

The online version contains supplementary material available at 10.1186/s10194-026-02440-w.

## Background

The first edition of the *International Classification of Headache Disorders* (ICHD-1), published in 1988 [1], did not use the term “chronic migraine” (CM). Instead, it recognized a group of patients with both migraine and tension-type headache (TTH), who were in a continuum from pure migraine through equal amounts of each to pure TTH.

But ICHD-1 acknowledged that “many parts of the document are based on the experience of the experts … in the absence of sufficient published evidence” [1], asserting that mistakes would be corrected in revisions to ICHD informed by clinical application and research.

The second edition (ICHD-2), published in 2004 [2], introduced 1.5.1 *Chronic migraine*, “a new diagnosis for those rare patients who fulfil the diagnostic criteria for migraine for 15 or more days a month without overusing medication.” The category “1.5” was reserved for *Complications of migraine*. These criteria were severely criticised even while they were being developed, mainly on the basis that very few patients met them [3, 4], but they survived the revision published in 2005 [5, 6]. In 2006, new criteria were proposed for A1.5.1 *Chronic migraine* – still a complication rather than a type of migraine [3] – with the prefix “A” (appendix) signifying that these were proposals for which evidential support was needed [2]. In essence, they redefined CM as “headache (tension-type and/or migraine) on ≥ 15 days/month”, fulfilling criteria for migraine on only ≥ 8 days/month.

These proposals did not introduce, but nonetheless gave later rise to, the terms “monthly migraine days” (MMDs) and “monthly headache days” (MHDs).

ICHD-3 beta, published in 2013 [7], brought 1.3 *Chronic migraine* into the main classification, accepting it as a type distinct from episodic migraine (classified as 1.1 *Migraine without aura* or 1.2 *Migraine with aura*). The notes explained: “The reason for singling out 1.3 *Chronic migraine* from types of episodic migraine is that it is impossible to distinguish the individual episodes of headache in patients with such frequent or continuous headaches” [7]. ICHD-3 beta complicated the criteria by allowing headache “believed by the patient to be migraine at onset and relieved by a triptan or ergot derivative” to be counted among the ≥ 8 MMDs [7]. Apart from this being the only instance in ICHD inserting a patient’s belief into a diagnostic criterion, it rather clouded the distinction between CM and medication-overuse headache (MOH) [8, 9]. The nosologic (or taxonomic) confusion introduced by reference to “headache (tension-type …)” in a diagnostic criterion for a type of migraine was recognized in ICHD-3 beta – if not fully resolved – by a change to “headache (migraine-like or tension-type-like)” [7]. What ICHD-3 beta did not do, and neither did ICHD-3 (the current, 3rd edition) [10], was challenge – or seek to justify – the thresholds of ≥ 8 MMDs and ≥ 15 MHDs. As with ICHD-1, there was an “absence of sufficient published evidence” to do either.

Two recent studies contributing to this debate stimulated the analyses presented here.

The first, a literature review, sought evidence that *high-frequency episodic migraine* (HFEM) should also be regarded as a clinical and/or biological entity distinct from both *low-frequency episodic migraine* (LFEM) and CM [11]. No new data were presented, but multiple differentials were identified, including attack features and comorbidities, work productivity, employment status and disability. A definition of HFEM was proposed for future testing: in essence, 8–14 MMDs but not CM [11]. In practice, the determining distinction between HFEM defined thus and most cases diagnosed as CM is in the number of non-migraine headache days (or, in ICHD-3 terminology, by days of “tension-type-like headache” [10]). As a basis for defining migraine types, this seems incoherent (perhaps supporting the original view of ICHD-1).

The second study, using population-based data from Norway, directly challenged this, or at least the distinction between HFEM (similarly defined) and CM [12]. It concluded that disease burden differentials between HFEM and CM were fully explained by frequency of MMDs. This placed HFEM and CM on a spectrum in which non-migraine headache days were unimportant [12].

The debate here has major significance for migraine nosology at a time when the 4th edition of ICHD (ICHD-4) is under development [13]. There is no doubt that this requires resolution, with regard both to LFEM *versus* HFEM and to HFEM *versus* CM. Our purpose here is to reconsider, and perhaps challenge, the entire notion of “episodic” and “chronic” migraine by examining the frequency spectrum of migraine in its entirety, from < 1 to 30 MMDs. Our question was not where the demarcating thresholds should be placed (inevitably arbitrarily, as eloquently argued by Fischer-Schulte and May [14]); rather, did population-based evidence on headache frequency, characteristics and attributed burden provide any support for putting thresholds anywhere?

## Methods

We used population-based data collected from adults with migraine, randomly selected through surveys conducted within the Global Campaign against Headache, all using similar methodology [15].

Clinical trial number: not applicable.

### Ethics

Ethics approvals and consents from participants in each contributing study were obtained in accordance with the Declaration of Helsinki [16] and local requirements [17–31].

### Study design

This was a meta-analysis of individual participant data (IPD) in the Headache-Attributed Restriction, Disability, Social Handicap and Impaired Participation (HARDSHIP) adult database [15]. Use of IPD in meta-analyses is methodologically superior to use of aggregate data [32], and, for our purposes here, provided the opportunity to regress variables against headache frequency (MMDs and MHDs) at individual level.

Data from > 47,000 participants from 24 countries (28 samples) are currently included in the HARDSHIP adult database [15]. We used data from 14 of these countries (15 samples): Benin [17], Cameroon [18], China [19], Ethiopia [20], India (Karnataka State [21] and North Capital Region/Delhi [22]), Lithuania [23], Mongolia [24], Morocco [25], Nepal [26], Pakistan [27], Peru [28], Russia [29], Saudi Arabia [30] and Zambia [31]. These samples were chosen because they were considered to be representative of the general adult populations (aged 18–65 years) from which they were drawn, and included the diagnostic data required to diagnose migraine in accordance with ICHD-3 [10].

Supplementary Table 1 shows detailed information on all samples currently in the database.

### Data acquisition

All contributing studies were cross-sectional and applied standardized methodology [33, 34]. In all but one, randomized cluster sampling ensured representativeness of the general population. Within each cluster, during unannounced visits at randomly selected households, one member of each aged 18–65 years, also randomly selected, was interviewed face-to-face. In Saudi Arabia, where culture precluded unannounced household visits, participants were contacted by cell-phone using random digit dialling (at the time of this survey, cell-phone coverage in Saudi Arabia exceeded 100%) [30].

All interviews were performed using the HARDSHIP questionnaire [34], translated into the local language(s) following a standardized translation protocol [35]. All data were collected between 2008 and 2020.

### Headache diagnoses

In the contributing studies, only participants reporting headache during the preceding year had been asked the subsequent diagnostic questions. Diagnoses were made algorithmically during data analysis, not by the interviewers.

For the purposes of this study, and contrary to all previous studies reporting HARDSHIP data, migraine and pMOH were not regarded as mutually exclusive diagnoses.

The algorithm derived diagnoses based on reported symptoms, with focus on the most bothersome headache when more than one headache type was reported. This process observed the hierarchical order of ICHD-3 [10]: first identifying definite migraine, then definite TTH, then probable migraine; this ensured that participants meeting criteria for definite TTH could not be included among those diagnosed with probable migraine. References in the analyses to “migraine” (without further specification) include both definite and probable migraine.

The algorithm also identified probable MOH (pMOH) as an additional diagnosis among those with migraine when headache (of any type) was reported on ≥ 15 days/month (H15+) along with acute medication overuse. We defined overuse as reported use on ≥ 10 days/month in countries where triptans, opioids and/or compound analgesics were considered readily available (Lithuania, Morocco, Russia and Saudi Arabia), and on ≥ 15 days/month in all others (Benin, Cameroon, Ethiopia, India, Mongolia, Nepal, Pakistan, Peru and Zambia). In the sample from China, we had no information on medication consumption and pMOH could not be diagnosed [36].

In cases of missing data regarding headache characteristics, the diagnostic algorithm applied neutral or conservative imputation rules with the effect of preferring probable rather than definite diagnoses [36].

Among participants from Nepal, photophobia had been reported by virtually everyone with headache, rendering it unhelpful in the differentiation between migraine and TTH [26]. Photophobia was therefore treated as missing for all Nepalese participants [26].

Enquiry in the contributing studies had also included headache on the day prior to interview (headache yesterday [HY]), and its duration when reported.

### Monthly headache and migraine days

All participants included in the further analyses had reported headache meeting criteria for migraine (definite or probable) as their only headache or as their most bothersome headache. Enquiry included frequency of this headache and of any headache, each recorded as continuous variables with days/month as units. The former provided the count of MMDs, the latter the count of MHDs. Both had a theoretical range of 0–30; in many of the studies, response options to both enquiries had included “every day”, which was interpreted as 30.

### Headache characteristics

Usual headache intensity, reported as “not bad”, “quite bad” or “very bad”, was recorded on an ordinal numerical scale as 1–3. For reasons explained in Discussion, we analysed duration of HY, when reported, rather than usual headache duration. We expressed this in hours, and recorded it as a continuous variable, with a theoretical range of 0–24 (response options included “all day”, which was interpreted as 24). Usual headache location (“unilateral” or “bilateral”), pain characteristic (“throbbing” or “pressing”) and aggravation by physical activity (“yes” or “no”) were also recorded. Nausea, vomiting, photophobia and phonophobia were all reported as usual accompanying symptoms or not, and recorded accordingly as “yes” or “no”.

### Quality of life

All contributing studies except those in Morocco [25], Saudi Arabia [30] and Zambia [31] recorded data on quality of life (QoL) using the World Health Organization 8-item Quality of Life assessment (WHOQoL-8) [37]. Each item was scored 1–5 (higher scores indicating better life quality), and the scores summed for an overall score in the range 8–40 [37], which was treated as a discrete variable.

### Impaired participation

All contributing studies recorded impaired participation attributed to headache using the Headache-Attributed Lost Time (HALT) indices [38]. Participants reported affected days in the preceding 3 months in the three domains of paid work, household work and social or leisure activities. According to recommended methodology, “less than half achieved” was equated to “nothing achieved”, counterbalanced by equating “more than half achieved” to “everything achieved” [38].

Only employed participants (including self-employed: i.e., those capable of losing days from paid work) were included in analyses of lost (paid) workdays. We had information on employment status in all contributing studies but Pakistan (*N* = 4,223) [29].

### Statistics

For the purpose of our analyses, we identified four different migraine groups, with different levels of diagnostic purity and inclusivity: (1) migraine (definite or probable) including those with concomitant pMOH; (2) migraine (definite or probable) excluding those with concomitant pMOH; (3) definite migraine including those with concomitant pMOH; and (4) definite migraine excluding those with concomitant pMOH.

For each of these four groups, the geom_smooth function (method=”loess”, formula=“y ~ x”) within the ggplot package of R was used to regress MMDs against age, headache characteristics (except headache intensity), WHOQoL-8 score, lost (paid) workdays, lost household days and lost social or leisure days. In addition, bar charts were used to investigate relationships with headache intensity, and histograms to display the distributions of MMDs. All these 15 analyses were then repeated for MHDs (a total of eight sets of 15 analyses).

We did not use imputation to replace missing data in the regressions: instead, participants missing data for a particular headache characteristic were excluded from that regression analysis.

We used RStudio version 2023.06.2 + 561 [39] for all analyses.

## Results

The total sample from the 15 contributing studies (14 countries) included 36,407 participants (16,955 [46.6%] males; 19,449 [53.4%] females; three participants missing gender information), with overall mean age of 37.5 years (SD = 13.0). Of these, 20,253 were reportedly employed (62.9% of the sample excluding Pakistan [employment data unavailable]).

A total of 10,266 participants (28.2%) fulfilled our criteria for migraine (5,310 [14.6%] definite, 4,956 [13.6%] probable), of whom 896 (2.5%) had concomitant pMOH (552 with definite migraine, 344 with probable migraine). HY was reported by 3,046 (888 males, 2,158 females) and 5,142 (56.8% excluding Pakistan; 2,288 males, 2,854 females) were employed.

Table 1 shows the essential characteristics of the sample.

**Table 1:** Essential characteristics of the sample

	Sample		
	Overall	Males^a^	Females^a^
**Sample size**, n (%)	36,407 (100)	16,955 (46.6)	19,449 (53.4)
**Age**^**b**^, mean±SD	37.4±13.0	37.8±13.0	37.2±12.9
18–25, n (%)	8,100 (22.3)	3,734 (22.0)	4,364 (22.5)
26–35, n (%)	9,686 (26.6)	4,310 (25.4)	5,376 (27.7)
36–45, n (%)	8,567 (23.5)	4,115 (24.3)	4,451 (22.9)
46–55, n (%)	5,784 (15.9)	2,752 (16.2)	3,032 (15.6)
56–65, n (%)	4,253 (11.7)	2,038 (12.0)	2,215 (11.4)
**Employed**^**c**^, n (%)	20,253 (62.9)	11,622 (77.5)	8,629 (50.2)
**Migraine**, n (%)	10,266 (28.2)	3,434 (20.3)	6,832 (35.1)
Definite, n (%) Probable, n (%) Concomitant pMOH, n (%) Headache yesterday, n (%)	5,310 (14.6) 4,956 (13.6) 896 (2.5) 3,046 (8.4)	1,595 (9.4) 1,839 (10.8) 205 (1.2) 888 (5.2)	3,715 (19.1) 3,117 (16.0) 691 (3.6) 2,158 (11.1)
**Country**, n			
Benin Cameroon China Ethiopia India, Delhi India, Karnataka Lithuania Mongolia Morocco Nepal Pakistan Peru Russia Saudi Arabia Zambia	2,400 3,100 5,041 2,385 2,066 2,329 572 2,041 2,575 2,100 4,223 2,149 2,025 2,316 1,085	1,231 1,407 2,561 1,057 737 1,141 236 812 1,038 861 1,957 1,065 960 1,442 450	1,169 1,693 2,480 1,328 1,329 1,188 336 1,229 1,535 1,239 2,265 1,084 1,065 874 635

pMOH: probable medication-overuse headache; ^a^Three participants were missing information on gender; ^b^17 participants were missing information on age; ^c^excluding Pakistan, where employment data were not available

**Fig. 1:**
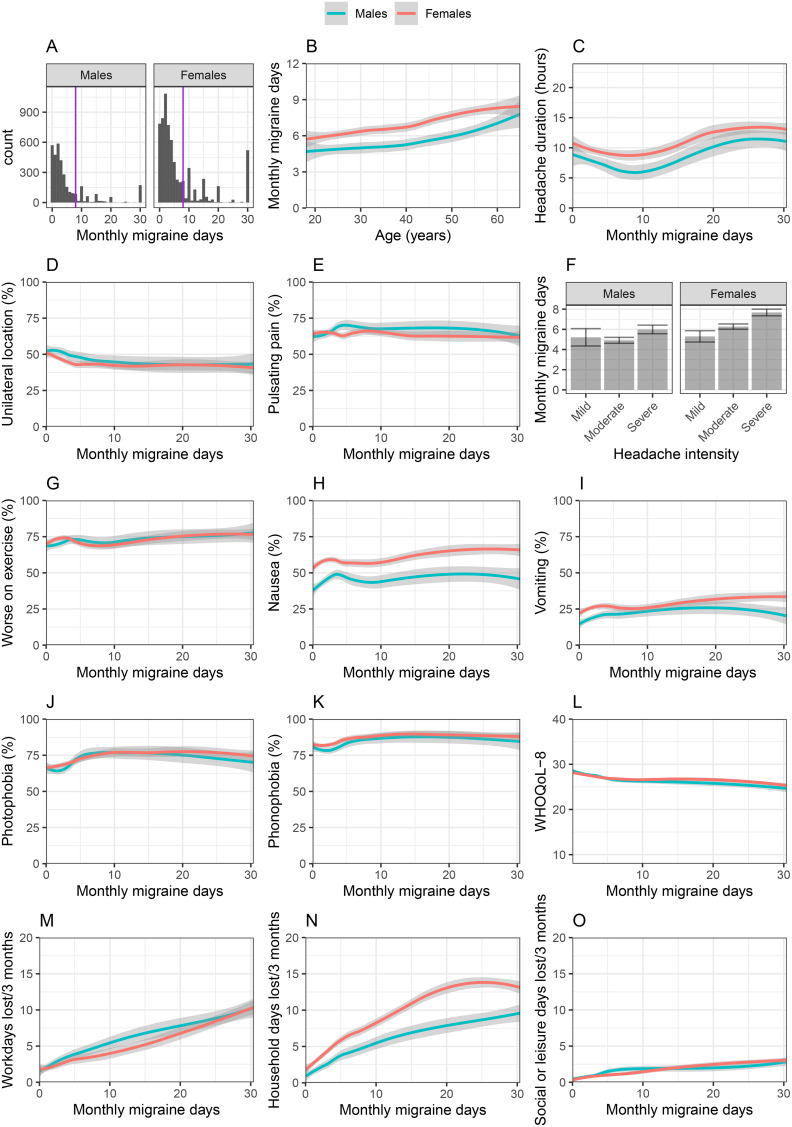
Migraine (definite+probable) including concomitant probable medication-overuse headache: monthly migraine days

**Fig. 2:**
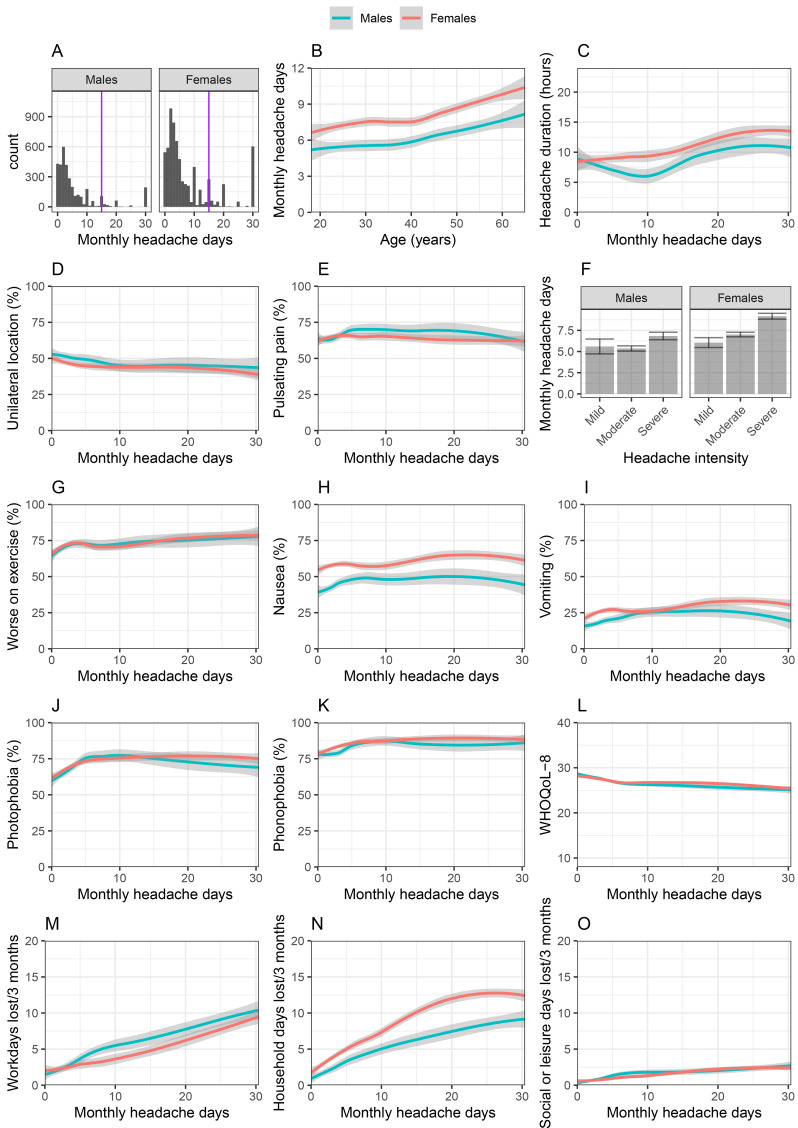
Migraine (definite+probable) including concomitant probable medication-overuse headache: monthly headache days

**Fig. 3:**
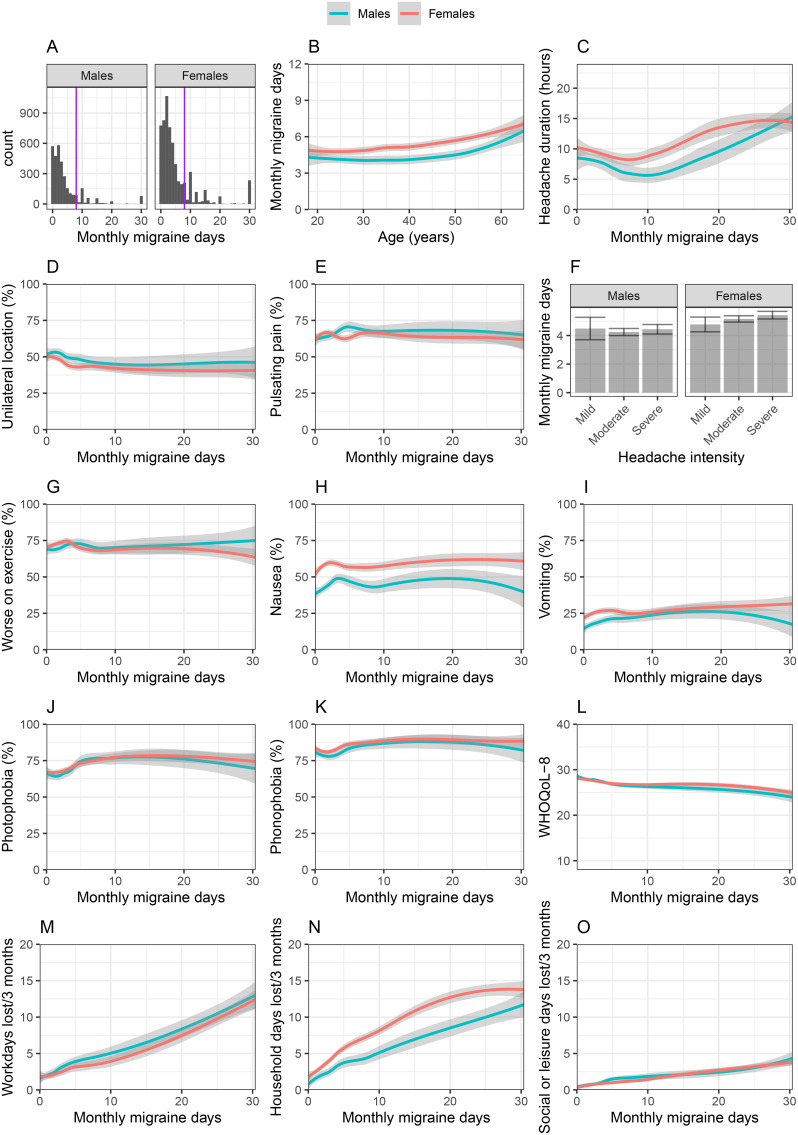
Migraine (definite+probable) excluding probable medication-overuse headache: monthly migraine days

**Fig. 4:**
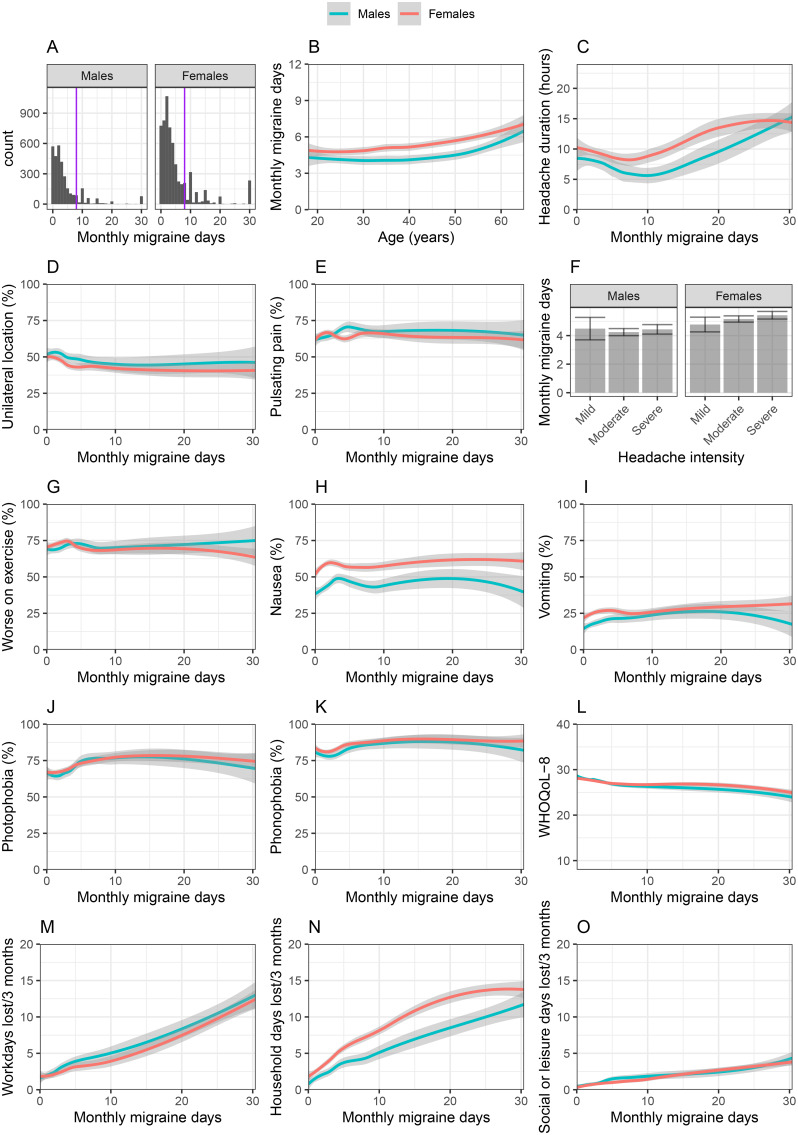
Migraine (definite+probable) excluding probable medication-overuse headache: monthly headache days

**Fig. 5:**
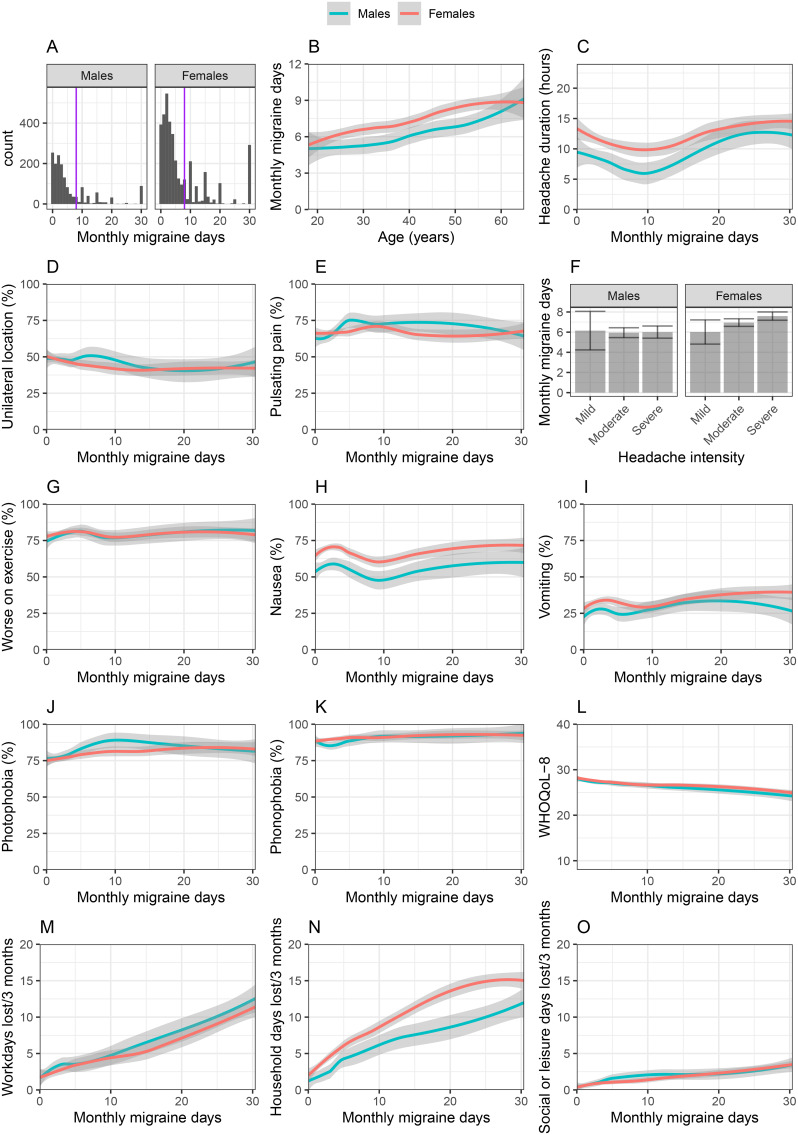
Definite migraine including concomitant probable medication-overuse headache: monthly migraine days

**Fig. 6:**
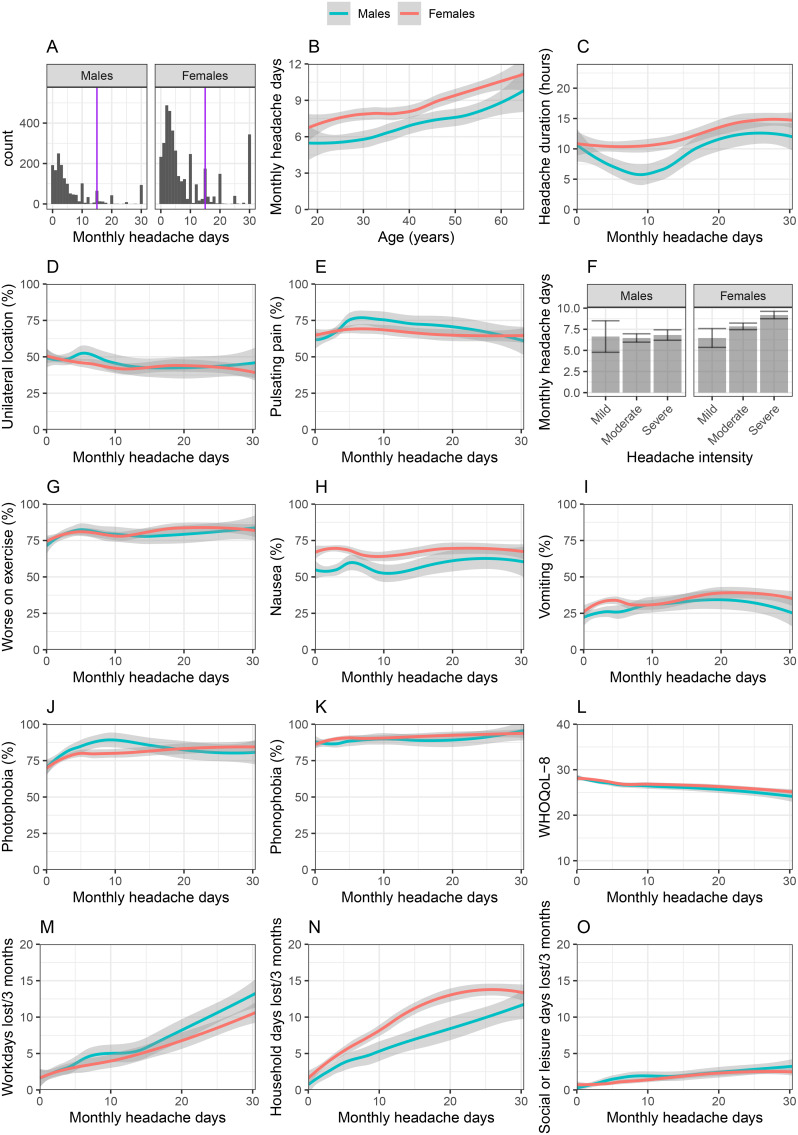
Definite migraine including concomitant probable medication-overuse headache: monthly headache days

**Fig. 7:**
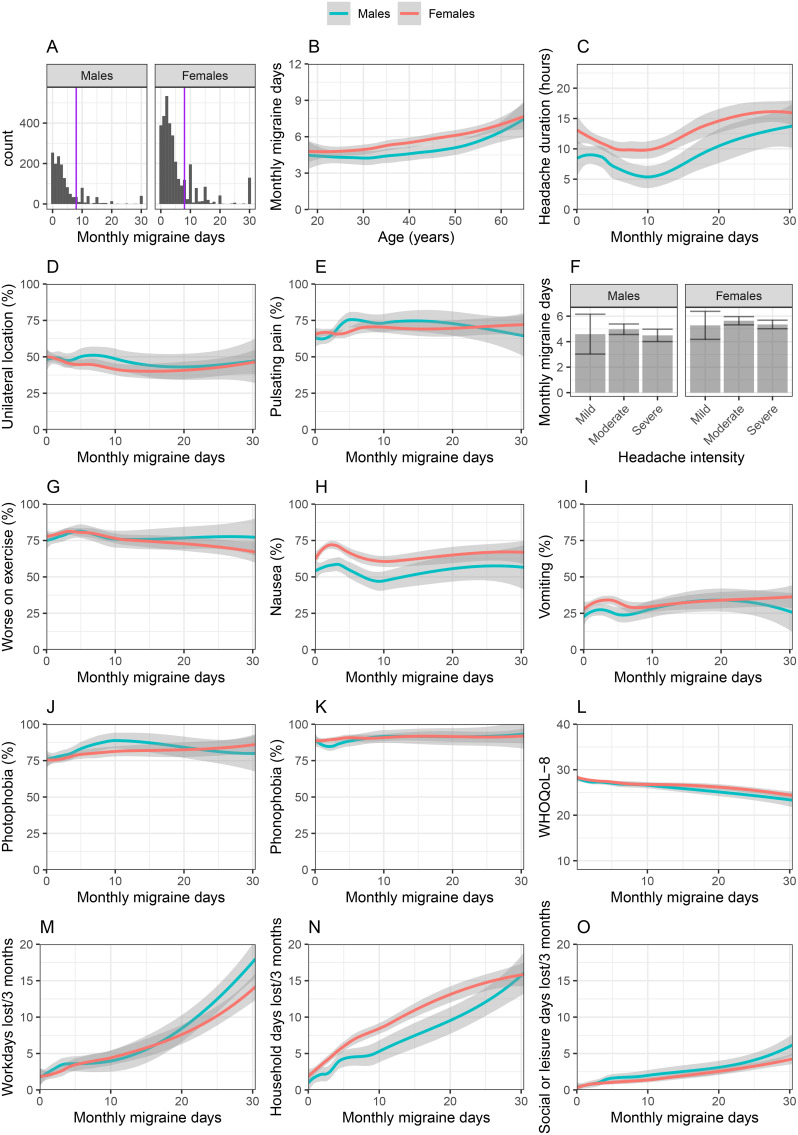
Definite migraine excluding probable medication-overuse headache: monthly migraine days

**Fig. 8:**
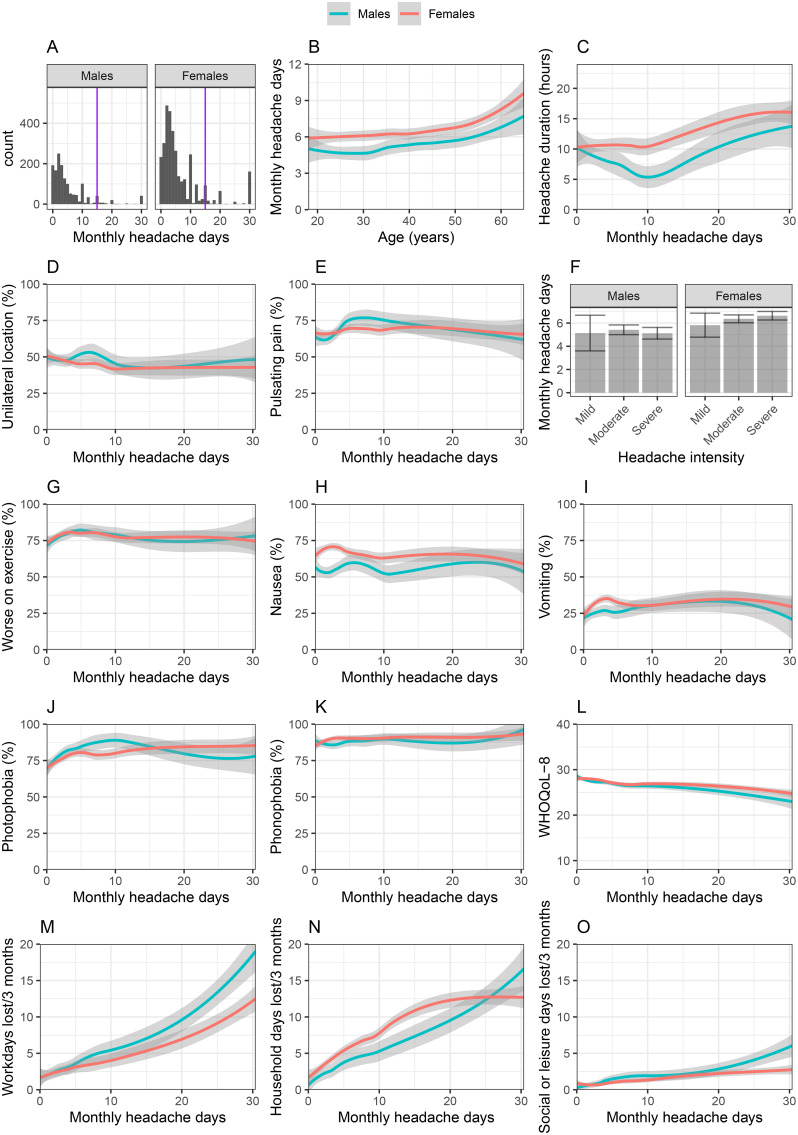
Definite migraine excluding probable medication-overuse headache: monthly headache days

Figures 1, 2, 3, 4, 5, 6, 7 and 8 (with odd numbers depicting MMDs and even numbers MHDs) show the eight sets of analyses across the four migraine groups, each figure displaying 15 charts coded A-O. Blue lines (in charts B-E and G-O) show estimates for males, red lines those for females. Error bars (in chart F) and grey shaded areas around the lines (in charts B-E and G-O) show 95% confidence intervals (CIs).

Supplementary Table 2 shows the numbers of participants with missing data for each variable of interest.

### Distributions of frequencies

Chart A in each figure is a histogram showing the distributions of MMDs and MHDs by gender. The purple vertical lines show the thresholds of MMDs (≥ 8) and MHDs (≥ 15) currently applied to identify CM [10].

All figures show the same: (1) heavily right-skewed distributions, within which most participants had relatively few MMDs and MHDs, both peaking at 3 days/month; (2) preferences for 0 or 5 as terminal digits, creating spurious smaller peaks; (3) relatively many reporting 30 MMDs or MHDs, probably reflecting a combination of terminal digit preference, the naturally capping effect (headache every day being the maximum possible)] and the inviting response option of reporting headache on “every day”; and (4) very similar distributions among males and females (smaller numbers among males reflecting the lower migraine prevalence).

### Relationships with age

Chart B in each figure shows variations in MMDs and MHDs with age, again by gender. In all figures, more or less perfectly linear positive relationships are seen, with females consistently reporting more MMDs and MHDs than males.

### Relationships with headache characteristics

Charts C-K in each figure show the relationships between headache characteristics and MMDs or MHDs.

With regard to HY duration (Chart C), among males a similar pattern is seen in all figures: a slight decline in HY duration as MMDs or MHDs increased from < 1 to 10, followed by an increase. Inclusion of those with pMOH resulted in flattening of the curves at around 25 MMDs or MHDs, not seen when pMOH was excluded. Among females, the pattern for MMDs is very similar (Figs. 1, 3, 5 and 7), albeit that females consistently reported slightly longer durations. For MHDs, on the other hand (Figs. 2, 4, 6 and 8), curves are generally flatter among females.

With regard to unilateral location (Chart D), all figures show the same flat line at around 50% in both males and females, indicating no variation in headache location with increasing frequencies.

With regard to pain characteristic (Chart E), throbbing pain was reported similarly by 70–75% of males and females, with minimal variation with MMDs or MHDs.

With regard to headache intensity (Chart F), among males there was little evidence of association between the three levels of intensity and frequencies of MMDs or MHDs (CIs largely overlapping), regardless of migraine group. Among females, however, linear relationships between intensity and frequency were present in those migraine groups that included pMOH, but they largely disappeared when pMOH was excluded.

With regard to aggravation by exercise (Chart G), all figures show the same flat line, indicating no variation with increasing frequency. The proportions reporting headache aggravation by exercise were slightly higher among those with definite migraine, but there were no differences between genders.

With regard to nausea (Chart H), all figures show this was more prevalent among females than males with migraine. In relation to frequency, similar patterns are seen for MMDs (Figs. 1, 3, 5 and 7): small humps, followed by troughs at about 10 days/month, then slight increases in nausea as frequencies increase above this. The curves are similar for MMDs and MHDs, while being flatter for MHDs.

With regard to vomiting (Chart I), the relationships with headache frequency mirrored those of nausea, with proportions reporting vomiting approximately half those reporting nausea.

With regard to photophobia (Chart J), increases (in most cases from 50 to 60% to 70–75%) were reported among those with migraine, with or without concomitant pMOH, as MMDs and MHDs increased to 5–10 days/month, before flattening at around 70% (Figs. 1, 2, 3 and 4). A similar pattern occurred for definite migraine (Figs. 5, 6, 7 and 8), but with higher proportions reporting photophobia (flattening between 75% and 80%). There were no differences between the genders.

Finally, with regard to phonophobia (Chart K), it was evident that, among both males and females, this was a very common experience, reported by about 80% of those with migraine and by about 90% of those with definite migraine (in each case with or without concomitant pMOH). No variations with frequency were apparent.

### Relationships with quality of life

Chart L shows the same in all figures: WHOQoL-8 scores slightly but steadily and linearly declined as MMDs or MHDs increased. Identical scores and relationships were reported by males and females.

### Relationships with impaired participation

With regard to lost workdays (Chart M), all figures show the same: very similar losses among males and females, and linear positive relationships with headache frequency (whether MMDs or MHDs), reaching > 10 lost workdays/3 months without any flattening. Definite migraine was associated with higher losses than probable; higher losses were also seen among the groups with pMOH excluded.

Lost household days (Chart N) increased linearly with MMDs or MHDs among males. Among females, lost household days were more, but plateaued as MMDs or MHDs reached 20–25 days/month and losses came close to 15 days/3 months. In both genders, inclusion or exclusion of pMOH and/or of probable migraine had little effect.

Lost days from social or leisure activities (Chart O) were considerably fewer than lost days from paid or household work, and did not differ between the genders. The relationships with MMDs and MHDs were to a large degree linear.

## Discussion

The purpose of this analysis was to examine whether population-based evidence supported the current distinction between EM and CM (or a distinction between LFEM and HFEM) or, alternatively, signalled that migraine would be better conceptualized as a single disorder expressed along a frequency spectrum. Analysing population-representative IPD from 10,226 migraineurs from 14 countries from all world regions, we found little to support the notion established in ICHD-3 [10] that EM and CM, defined by MHDs or MMDs, were distinct entities. Instead, across all domains examined — headache characteristics, associated symptoms, QoL and impaired participation — relationships with headache frequency were predominantly linear, continuous, and without natural inflection points. These findings argue strongly for a spectrum model of migraine, and have important implications for migraine nosology and ICHD-4.

Most headache features regarded as “migraine-defining” were strikingly stable across the entire frequency spectrum. Unilateral location, throbbing pain, aggravation by physical activity and phonophobia showed virtually no variation with MHDs or MMDs. In the few cases where variations were observed (nausea and photophobia), these were modest (within 10–15% points) and did not signal any transition to a qualitatively different disorder.

The relationship between frequency and duration of HY was one of the few non-stable and clearly non-linear patterns observed, but is biologically and clinically plausible. The initial decline in duration as frequency increased to around 10 days/month might be explained by improved acute management, with higher attack frequency motivating a drive for more effective treatment. It might also reflect longer refractory periods (hence lower frequency) following longer attacks. The subsequent increase in duration at higher frequencies (> 10 days/month) is readily explained by the tendency of longer-lasting headaches to extend into subsequent days, thereby inflating frequency counted in days/month rather than attacks/month. Importantly, this pattern does not imply a categorical shift in disease type but, rather, an interaction between headache duration and headache days within a single-disorder continuum. Awareness of this interaction, and the desire to minimize its effect, prompted us to analyse duration of HY (naturally capped at 24 h) and not usual headache duration (often > 24 h), but its effect could not be fully eliminated (duration of HY is naturally and inevitably correlated with usual headache duration).

WHOQoL-8 scores declined steadily and linearly with increasing frequency, with identical scores in males and females. Despite the cross-cultural variability in baseline scores and the lack of meaningful units, WHOQoL-8 has consistently demonstrated sensitivity to headache-attributed burden in previous studies [40–44]. Our findings here confirm frequency as a strong predictor of reduced QoL, but provide no evidence of a frequency threshold beyond which QoL deteriorates disproportionately.

Similarly, lost workdays increased linearly with frequency across all analyses, with no evidence of inflection points. The absence of gender differences is unsurprising since analyses were restricted to employed participants, effectively correcting for gender-related differences in employment rate. Lost household days among females plateaued at around 15 days/3 months, probably reflecting a saturation effect imposed by household demands (there is a limit to how many days of household chores are planned, and, probably, also a limit to how many can be abandoned) rather than a change in disease biology. In males, among whom baseline participation in household chores was generally lower, losses were smaller and increased linearly without reaching a plateau (probably not reaching saturation). Losses from social and leisure activities were also broadly linear, while comparatively small, probably because fewer days, generally, are set aside for these activities.

In summary, increasing MMDs and MHDs translate into increasing burden in a continuous manner, without stepwise changes. Measures of burden are consistently supportive of the concept of migraine as a single disorder expressed along a frequency spectrum, with no suggestion of distinct migraine types defined by frequency.

### Migraine days versus headache days

A notable finding was the close correspondence, with strikingly similar results in all cases, between analyses based on MMDs and those based on MHDs. This might not be expected: on non-migraine headache days and on MMDs, participants in the various surveys had expressly recognized different headache types, the former most likely to be TTH, which is usually considered to be relatively non-burdensome [45]. But the reality was that, in these population-based surveys, most participants with migraine (69.4%) reported only one headache type (so MMDs = MHDs), while non-migraine headache days were relatively few among those with more than one type. The implication is that the distinction between MMDs and MHDs adds little explanatory power to the analysis of burden across the frequency spectrum. This is a salient observation, because it directly challenges the nosologic importance placed on non-migraine headache days in distinguishing HFEM from CM, as proposed by some [11]. Our findings are consistent with the Norwegian population-based study showing that burden differences between HFEM and CM (as defined) are explained by MMDs alone [12].

### The effect of diagnostic purity

Inclusion of participants with concomitant pMOH increased the number with higher headache frequencies (≥ 15 days/month) but had remarkably little impact on the regression patterns. Similarly, restricting analyses to definite migraine did not materially alter our findings. This robustness across levels of diagnostic purity (or, conversely, of inclusivity) strengthens our interpretation that the observed (continuous) relationships are intrinsic to migrainous headache, and are not artefacts of diagnostic overlap or misclassification.

One notable exception was headache intensity among females, who reported a positive relationship with frequency only when concomitant pMOH was included. This is consistent with several studies showing pMOH to be associated with higher pain intensity than migraine [40–44], although this has not been a universal finding. In males, among whom pMOH is less prevalent [36], we found no positive relationship between intensity and frequency.

### Frequency thresholds and the EM–CM distinction

The thresholds for diagnosis of CM of ≥ 15 MHDs along with ≥ 8 MMDs have been defended as reasonable and sufficient, notably by Fischer-Schulte and May, who argued that these thresholds, while inherently arbitrary, were pragmatically useful, and that distinction of CM from EM was *clinically* meaningful [14]. Our data do not wholly contradict this view, but challenge the assumption that any particular threshold corresponds to a qualitative change in disease expression such that it places a diagnostic separator between two disease entities. Thresholds, wherever set, are merely markers placed along linear and continuous relationships, while the histograms of both MMDs and MHDs show no steps or clustering other than expected artefacts — terminal digit preferences, and an accumulation at 30 days/month reflecting the upper bound of the scale. This perception is consistent with the opinions of Ishii et al. [46] and Newman et al. [47]: that the 15-day threshold does not reflect a detectable change in disability across the migraine frequency spectrum.

Importantly, our analyses extend this debate beyond disability alone. Across dozens of analyses, of headache characteristics, QoL and measures of impaired participation, the dominant visual impression is of straight lines, with any deviations from linearity explicable without invoking distinct disease entities. Frequency behaved throughout as a continuous predictor, not as a discriminator between putative migraine types.

### Comorbidities and tractability

Multiple authors have argued that EM and CM are distinct on the basis of differing comorbidities and tractability [48–51]. We acknowledge both this argument, and that our dataset did not include information on conditions such as anxiety, depression and more generalized pain disorders that are known not only to be comorbid with migraine [51, 52] but also more prevalent among those diagnosed with CM [51, 53]. But this does not invalidate our opposing argument, or even contradict it. It is equally plausible that these comorbidities, and intractability, are, simply, associated (causally or consequentially) with increasing symptom burden, and do not signal categorical biological differences between high-frequency and lower-frequency migraine.

### Implications for migraine classification and ICHD-4

Had these population-based data been available during the gestation and birth of ICHD-3, the concept of CM as an entity distinct from EM might not have emerged as it did.

Yet, while there is no support from our population-based data for nosologic recognition of frequency-defined migraine types, distinctions based on frequency of MHDs might still serve clinical purposes – in treatment plans, selection of patients for clinical trials of treatments, headache service organization and provision, or reimbursement frameworks. If so, where should non-arbitrary thresholds be set? Certain interventions — most notably onabotulinumtoxin A — have demonstrated efficacy in patients meeting criteria for CM but not EM [54], but this may simply reflect trial design, patient selection and the greater margin for measurable change offered by higher baseline frequencies rather than biological discontinuity. Identifying thresholds predictive of therapeutic response, if any exist, requires data not available in the present analyses.

Two MMD thresholds appear clinically purposeful: ≤3 MMDs would not usually be considered for preventative therapy; ≥10 MMDs signals risk of medication overuse and development of MOH. In accordance with these, *low-frequency*, *high-frequency* and *very-high-frequency* might be attached as *qualifiers* to any diagnosis of migraine.

A major constraining factor in disease classifications, including ICHD, is that significant changes to diagnostic criteria create confusion in the literature. This already exists in relation to CM, for which the ICHD criteria were never universally adopted. The term “chronic” is used infelicitously with regard to migraine; now would be a good time to abandon it.

### Strengths and limitations

The principal strengths of this study were its use of population-based data derived from diverse countries and cultures, the large sample size (*N* = 36,407, of whom 10,226 had migraine), and the use of IPD enabling regression analyses across the full frequency spectrum. Limitations included the cross-sectional nature of the data and reliance on self-reported frequency and characteristics. We lacked information on treatment responses, which might (or might not) have been informative.

## Conclusions

Our findings provide strong population-based evidence that migraine, when considered in relation to headache frequency, characteristics and attributed burden, is better understood as a spectrum disorder rather than as two or more entities distinguished categorically by frequency. The current ICHD-3 thresholds for CM do not align with detectable step-increments, or other changes, in headache characteristics or burden. As ICHD-4 is developed, consideration should be given to reframing migraine classification according to frequency in a way that reflects continuity rather than categorical separation.

## Supplementary Information

Below is the link to the electronic supplementary material.

Supplementary Material 1

## Data Availability

The original data are held at Norwegian University of Science and Technology, Trondheim, Norway. Anonymised data are available on request for academic purposes, in line with the policy of the Global Campaign against Headache.

## Abbreviations

CI: Confidence interval
H15+: headache on ≥ 15 days/month
HALT: headache-attributed lost time (indices)
HARDSHIP: Headache-Attributed Restriction, Disability, Social Handicap and Impaired Participation (questionnaire)
HY: headache yesterday
ICHD: International Classification of Headache Disorders
LTB: Lifting The Burden
MHD: monthly headache day
MMD: monthly migraine day
MOH: medication-overuse headache
NorHEAD: Norwegian Centre for Headache Research
pMOH: probable MOH
QoL: quality of life
SD: standard deviation
TTH: tension-type headache
WHOQoL-8: World Health Organization 8-item Quality of Life (assessment)
