# Supplementary material for: Is migraine a spectrum disorder? Questioning the concept of “episodic” and “chronic” migraine using population-based data from 10,226 adults with migraine from 14 countries
Source: J Headache Pain. 2026 Jun 26;27(1):163. doi: 10.1186/s10194-026-02440-w (PMC13309956; doi:10.1186/s10194-026-02440-w)
Supplement: Supplementary file 1 — Supplementary Material 1 [file 10194_2026_2440_MOESM1_ESM.docx]

**Supplementary Table 1. Design and characteristics of all studies currently included in the adult Headache-Attributed Restriction, Disability, Social Handicap and Impaired Participation (HARDSHIP) database**

| **Study** | **Data collection (year)** | **Underlying population** | **Sampling method** | **Sample size**  **(N)** | **Participating proportion** | **Engagement** | **Interviewers** | **Included in this meta-analysis** |
| --- | --- | --- | --- | --- | --- | --- | --- | --- |
| Austria* | 2009 | Clinic-based, patients consulting GPs or neurologists for any reason | Random sample uncorrected for demographics | 519 | Indeterminable | Self-administered questionnaire | None, handed questionnaire at consultation | No |
| Benin | 2020 | General population from whole country | Random sample corrected for demographics | 2,400 | 94.1% | Face-to-face interviews | Trained interviewers with medical/research background | Yes [17] |
| Cameroon | 2019 | General population from whole country | Random sample corrected for demographics | 3,100 | 93.3% | Face-to-face interviews | Trained interviewers with medical/research background | Yes [18] |
| China | 2008-2009 | General population from whole country | Random sample corrected for demographics | 5,041 | 94.1% | Face-to-face interviews | Neurologists | Yes [19] |
| Ethiopia | 2014 | General population from whole country | Random sample corrected for demographics | 2,385 | 99.8% | Face-to-face interviews | Trained nurses | Yes [20] |
| France | 2009 | Clinic-based, patients consulting GPs for any reason | Random sample uncorrected for demographics | 680 | 36.5% | Self-administered questionnaire | None, handed questionnaire at consultation | No |
| Germany | 2009 | General population from part of the country | Random sample corrected for demographics | 318 | 10.6% | Self-administered questionnaire | None, contacted by regular post | No |
| India, Delhi region | 2018-2019 | General population from part of the country | Random sample corrected for demographics | 2,066 | 67.9% | Face-to-face interviews | Trained research officers | Yes [22] |
| India, Karnataka state | 2009 | General population from part of the country | Random sample corrected for demographics | 2,329 | 92.6% | Face-to-face interviews | Trained research officers | Yes [21] |
| Ireland* | 2009 | Members of patient organization | Total defined population | 225 | Indeterminable | Self-administered questionnaire | None, handed questionnaire at consultation | No |
| Italy* | 2009 | General population from part of the country | Stratified (age, sex, habitation), random sample | 487 | 13.9% | Self-administered questionnaire | None, contacted by regular post | No |
| Lithuania* | 2009-2010 | General population from part of the country | Stratified (age, habitation), random sample | 572 | 50.4% | Face-to-face interviews | Trained medical students | Yes [23] |
| Luxembourg* | 2008-2009 | General population from whole country | Stratified (age, gender, habitation), random sample | 1,825 | 28.2% | Self-administered questionnaire | None, contacted by regular post | No |
| Mali** | 2021 | General population from whole country | Random sample corrected for demographics | 2,105 | 99.4% | Face-to-face interviews | Trained interviewers | No |
| Mongolia | 2017 | General population from whole country | Random sample corrected for demographics | 2,041 | 98.3% | Face-to-face interviews | Trained neurologists | Yes [24] |
| Morocco (population sample) | 2019 | General population from whole country | Random sample corrected for demographics | 2,575 | 90.0% | Face-to-face interviews | Trained medical students | Yes [25] |
| Morocco (Fès sample) | 2019 | Selected population from part of the country | Convenience sample not corrected for demographics | 899 | Indeterminable | Face-to-face interviews | Trained medical students | No |
| Nepal | 2013 | General population from whole country | Random sample corrected for demographics | 2,100 | 99.6% | Face-to-face interviews | Trained health workers | Yes [26] |
| Netherlands (patient sample)* | 2009 | Members of patient organization | Random sample not corrected for demographics | 430 | Indeterminable | Self-administered questionnaire | None, handed questionnaire at consultation | No |
| Netherlands (population sample)* | 2010 | General population from whole country | Stratified (age, gender, habitation, education social status), random sample | 2,414 | Indeterminable | Self-administered questionnaire | None, contacted through internet | No |
| Pakistan | 2010 | General population from whole country | Random sample corrected for demographics | 4,223 | 98.5% | Face-to-face interviews | Trained non-medical interviewers | Yes [27] |
| Peru | 2019 | General population from whole country | Random sample corrected for demographics | 2,149 | 90.1% | Face-to-face interviews | Trained health workers | Yes [28] |
| Russia | 2008 | General population from whole country | Random defined sample | 2,025 | 74.3% | Face-to-face interviews | Trained non-medical interviewers | Yes [29] |
| Saudi Arabia | 2012 | General population from whole country | Random defined sample | 2,316 | 86.5% | Telephone interviews | Trained research coordinators with health background | Yes [30] |
| Spain (patient sample)* | 2009 | Members of patient organization | Random sample uncorrected for demographics | 264 | 58.8% | Self-administered questionnaire | None, handed questionnaire at consultation | No |
| Spain (workplace sample)* | 2009 | Workplace: postal service employees | Total defined population | 968 | 58.8% | Self-administered questionnaire | None, contacted by internal post | No |
| United Kingdom* | 2009 | Clinic-based, patients consulting GPs for any reason | Random sample uncorrected for demographics | 99 | 17.6% | Self-administered questionnaire | None, handed questionnaire at consultation | No |
| Zambia | 2012 | General population from whole country | Random sample corrected for demographics | 1,085 | 95.7% | Face-to-face interviews | Trained health workers | Yes [31] |

*Part of the Eurolight study
**Data on migraine not available

**Supplementary Table 2. Numbers of participants with missing data for the variables of interest**

| **Variable** | **N (%)** |
| --- | --- |
| Monthly headache days | 36 (0.4) |
| Monthly migraine days | 36 (0.4) |
|  |  |
| Duration of headache yesterday | 172 (5.6)^a^ |
| Headache location | 47 (0.5) |
| Pain characteristic | 29 (0.3) |
| Headache intensity | 35 (0.3) |
| Aggravation with physical activity | 177 (1.7) |
| Nausea | 24 (0.2) |
| Vomiting | 71 (0.7) |
| Photophobia | 177 (1.9)^b^ |
| Phonophobia | 100 (1.0) |
|  |  |
| WHOQoL-8 | 28 (0.3)^c^ |
|  |  |
| Lost workdays | 153 (3.0)^d^ |
| Lost household days | 1,269 (12.4) |
| Lost social or leisure activities | 1,103 (10.8) |

WHOQoL-8: World Health Organization 8-item Quality of Life assessment; ^a^Among those reporting headache yesterday; ^b^The sample from Nepal excluded because photophobia was treated as missing in this study; ^c^The samples from Morocco, Saudia Arabia and Zambia excluded because WHOQoL-8 was not captured in these studies; ^d^Among employed individuals
